# Association between Academic Performance, Physical Activity, and Academic Stress in Compulsory Secondary Education: An Analysis by Sex

**DOI:** 10.3390/children11101161

**Published:** 2024-09-25

**Authors:** Dilan Galeano-Rojas, Marina Cuadros-Juárez, Borys Bismark León Reyes, Mónica Alexandra Castelo Reyna, Claudio Farías-Valenzuela, Pedro Valdivia-Moral

**Affiliations:** 1 Department of Musical, Plastic and Corporal Expression Didactics, Faculty of Educational Sciences, Universidad de Granada, 18071 Granada, Spain; dagaleanor@correo.ugr.es (D.G.-R.); mariinacj10@correo.ugr.es (M.C.-J.); 2 Facultad de Educación, Universidad Estatal de Milagro UNEMI, Machala 070201, Ecuador; bleonr@unemi.edu.ec; 3 Faculty of Computer Science and Electronics, Escuela Superior Politécnica de Chimborazo, Riobamba 060105, Ecuador; monica.castelo@espoch.edu.ec; 4 Escuela de Ciencias de la Actividad Física, Universidad de Las Américas, Santiago 9170022, Chile; cfaria46@edu.udla.cl

**Keywords:** academic stress, academic performance, physical activity, gender, secondary education

## Abstract

Objectives: The main objective of this study is to analyze the relationships between academic performance, physical activity, and academic stress in secondary education students, while the secondary objective is to establish differences by gender in the physical activity and academic stress levels of secondary students based on academic performance. Methods: The sample was composed of students from both sexes who attended public institutions. Data collection was conducted applying an ad hoc questionnaire for academic performance, the PAQ-C questionnaire for physical activity, and the QASSE questionnaire for academic stress. Data analysis was performed using descriptive statistics: Spearman’s correlation coefficient was used for associations, while comparisons were conducted via the Mann–Whitney U test and Kruskal–Wallis H test. Results: The results show that academic stress is negatively correlated with physical activity and academic performance. Men present significantly higher values in physical activity, while women present higher mean values in general academic stress and the academic overload dimension. Lastly, regarding academic performance, significant differences were observed in the family pressure dimension, with students who perform better academically presenting lower mean values in this dimension of academic stress. Conclusions: In conclusion, the more the general academic stress, the lower the physical activity levels and academic performance. In addition, physical activity appears as a potential coping strategy for academic stress, and its influence on academic performance should be further studied in secondary education.

## 1. Introduction

Currently, the health and wellbeing of adolescents have become topics of special interest at the global level [1,2,3]. The adolescence stage implies significant behavioral changes that may lead to unstable and unhealthy behaviors attributed to adherence to pernicious habits such as alcohol or drug consumption, a poor diet, and sedentarism [4,5]. Therefore, adolescence is a key stage to establish a healthy lifestyle that reflects on other dimensions of life such as education and professional perspectives, which will, in turn, have implications at the physical, psychological, and emotional levels [2,6,7].

Sedentarism has become one of the most widespread risk factors in the world’s population [8,9]. It is estimated that more than 50% of adolescents exhibit sedentary behaviors and have a physically inactive lifestyle [10,11,12]. In addition, according to Barth et al. [13], physical inactivity in children and youngsters is accompanied by an increase in mental health problems. More than 14% of children in the world present these conditions [14], which can impact their quality of life in the short, medium, and long term, as this is a critical period for their development [5,15,16,17].

In particular, the transition to secondary education is, for most students, a stage riddled with anxiety and stress due to the academic demands and the new educational, social, and physical environment to which they need to adapt [18,19]. According to Naranjo [20] and Marcial et al. [21], the increase in stress levels can cause students physical, mental, and emotional health problems, reducing their self-esteem and affecting their personal development. In this context, academic stress consists of a reaction of students to school stimuli or demands considered stressors—i.e., exams, homework and task overloads, family pressure, or their own expectations—which affect both their wellbeing and academic performance [22,23,24].

In this sense, academic stress is recognized as a risk factor and a problem that school centers should prevent by fostering a positive and healthy environment [25]. Furthermore, considering that the sources of academic stress in general cannot be avoided, generating suitable coping strategies becomes necessary [26], mainly because of the relationship between academic stress and academic performance, which is key at the personal, professional, family, and institutional level [27,28]. Academic performance is understood as students’ achievement and progress in terms of learning, based on the educational objectives established during the school stage [29]. According to Scrimin et al. [26] and Colunga-Rodríguez et al. [30], the close link between academic stress and academic failure can generate higher levels of frustration and anxiety that, in turn, are related to worse academic performance. This becomes even more evident in higher education stages, as reflected by a higher school failure rate [31,32].

In this sense, the study of factors that affect academic performance, such as academic stress, has gained growing interest at the social and research level, as the influence of parents, teachers, and some sectors of society are decisive [28,33]. In this line, various studies indicate that the practice of regular physical activity (PA) can positively contribute to the academic performance and psychological wellbeing of students [19,34,35,36]. PA is understood as any body movement produced by the skeletal muscles that implies spending energy and allows for performing daily activities in the environment [37]. More physically active students with high levels of stress have been found to present less stress-induced diseases, while students with a more sedentary lifestyle and high levels of stress are more vulnerable to various diseases [11,38,39]; therefore, PA may have a protective effect against stress and other associated diseases [25].

In fact, Wunsch et al. [24] maintain that PA acts as a moderator variable between stress and academic performance, as it has positive effects on memory, reaction time, creativity, intelligence, and the synthesis process as a consequence of higher levels of oxygen in the brain, and it improves cognitive function [13,40]. In addition, strong factors such as an involvement with teachers and classmates and the possibility of practicing PA at school and outside school are associated with lower stress levels, with PA being an effective coping strategy for frustration that improves the mood and reduces anxiety levels [41], as well as facilitating adherence to healthy life behaviors such as good sleep and diet, which are in turn associated with better academic performance [36,42].

In this sense, considering that adolescents spent a high number of hours at school, and that the stress derived from various demands is a factor that affects academic performance [16], the relationship between stress and the lifestyle choices related to the practice of PA should be identified, as according to Visier-Alfonso et al. [42], the mechanisms behind this influence are still unclear. Contextual factors, such as the academic periods during which stress is assessed, the type of stress, and the nature of the PA interventions (exercise, training, sports, etc.), as well as the bidirectional or tridirectional relationships explored in the investigation of stress and its connection to other psychosocial variables [15,24,36], highlight the need for further research on this topic at the secondary education level. Therefore, the main objective of this study is to analyze the relationships between academic performance, PA, and academic stress in secondary students, while the second objective is to establish differences by gender in the PA and academic stress levels of students based on academic performance.

## 2. Materials and Methods

### 2.1. Participants

The sample was composed of 310 students from mixed public Compulsory Secondary Education (ESO, in Spanish) institutions from the Spanish provinces of Granada, Jaén, and Málaga. The sampling was non-probabilistic and by convenience, as participants were selected according to the characteristics of the study [43].

Regarding the characteristics of the sample, the mean age of the participants was 13.9 years old (SD = 1.22), 169 were men (54.5%), 125 were women (40.3%), and 16 did not specify a sex (5.2%). In addition, 82 participants were in their 1st year of ESO (26.5%), 74 in their 2nd year (23.9%), 96 in their 3d year (31%), 53 in their 4th year (17.1%) and 5 did not specify their year (1.6%).

### 2.2. Design

The study employed a quantitative research approach with a non-experimental and a descriptive cross-sectional design, as the variables were not manipulated, and the data collection was conducted at a specific time and only once in order to describe the phenomena, contexts, and facts analyzed and explain the trends in the population under study [43].

### 2.3. Instruments and Variables

A Likert scale was used as it enabled the measurement of different dimensions, such as the beliefs, thoughts, feelings, and behaviors of the participants, through various questions. The variables and measurement instruments validated for the population under study are introduced below:

- Sociodemographic characteristics

An ad hoc questionnaire was employed to collect the following sociodemographic data: age, sex, type of school attended, country of origin, and grade.

- Academic performance

An ad hoc questionnaire was created as in previous studies [13,44]. The mathematics, physical education, language, and English grades of the last term were considered to establish general academic performance. The measurement scale is the same used to grade students at school centers, i.e., ranges from 1 to 10. In addition, the criterion adopted to establish the academic performance level of students was “low” for grades from 0 to 6, “medium” for grades from 7 to 8, and “high” for grades from 9 to 10, as in other studies [30,45].

- Physical activity

The Spanish version of the PAQ-C questionnaire was applied [46]. This instrument calculates the PA of students during the last seven days through 10 questions, of which 9 refer to the level of PA and one to the presence of pathologies or events that impeded conducting PA during the last week. The response scale generates scores from 1 to 5, with higher scores indicating higher levels of PA: The internal consistency of the instrument presented in this study was α = 0.91, and it obtained α = 0.83 after validation.

- Academic stress

The Questionnaire on Academic Stress in Secondary School (QASSE) was employed [22]. The instrument is composed of 24 items presented on a Likert scale, where 1 equals “no stress at all” and 5 “a lot of stress”. The questionnaire measures the following dimensions: academic overload and school performance (items 1, 4, 5, 7, 10, 16, 18, 20, and 23), interaction with classmates, (items 2, 3, 6, 8, 9, 17, and 24), family pressure (items 15, 19, 21, and 22) and future prospects (items 11, 12, 13, and 14). The internal consistency of the questionnaire after its validation was α = 0.92, and in this study, it reached α = 0.91.

### 2.4. Procedure

A literature review was first conducted to have an overview of research on this topic. Then, the objective of the study was established, and the instruments for data collection were selected. Subsequently, the management team from the ESO institutes was contacted and informed about the purpose of the study through an informative letter, as well as about the approval of the same by the Ethics Committee of Universidad de Granada under the code 3324/CEIH/2023. Upon acceptance, schools were requested to send informed consent forms to the guardians or parents of students. In addition, the students’ families were informed about the objective of the students’ participation, clarifying that this was completely voluntary, anonymous, and confidential, and that students could abandon the study at any time in line with the recommendations of the Declaration of Helsinki [47].

For data collection, participants were given paper questionnaires. When answering them, students were accompanied by members of the research team, who could answer any questions or solve any problems that might arise. Lastly, after data collection, the data were statistically analyzed to obtain the results, discuss them, and establish conclusions based on the objectives of the study.

### 2.5. Statistical Analysis

The data normality was compared using the Kolmogorov–Smirnov test. Descriptive statistics are presented as mean, standard deviation, frequency, and percentages. The internal consistency of the questionnaires used was calculated via Cronbach’s alpha. Based on normality, it was decided that non-parametric tests would be used for inferential statistics. To determine the degree of correlation between academic performance, physical activity, and academic stress, Spearman’s correlation test was employed. Subsequently, Mann–Whitney’s U test was used to compare means by sex, while Kruskal–Wallis’s H test was selected for academic performance, calculating the size effect through Hedges’ g and eta squared, respectively. The statistical program used was SPSS V27 (Inc., IBM Corp., Armonk, New York, NY, USA). A significance of <5% was adopted.

## 3. Results

The results of this work are presented below in the same order as the established objectives and based on the statistical tests mentioned above. The mean and standard deviation values of the variables under study are shown in Table 1.

PA is observed to present an M = 2.66, i.e., moderate PA levels. In academic performance, M = 6.87 is on a “medium” scale value, with an SD = 1.62, which indicates some variability among participants. Regarding general academic stress, an M = 2.88 value is observed, with participants showing higher academic stress levels in the academic overload dimension (M = 3.34) and lower ones in the classmate interaction dimension (M = 2.38).

In Table 2, a descriptive analysis is presented based on the academic performance levels, i.e., low, medium, and high academic performance.

After analyzing the percentages, 30% of the students present a low academic performance, while most participants (46.4%) have a medium academic performance. In turn, the smallest percentage (23.6%) corresponds to students with a high academic performance.

Table 3 presents the results of the correlation analysis between variables through Spearman’s coefficient.

Initially, PA is observed to be negatively related to general academic stress (*p* = 0.049, rho = −0.150), i.e., the higher the PA level, the lower the academic stress levels. In turn, academic performance is negatively correlated to general academic stress (*p* = 0.016, rho = −0.169) and the dimensions of family pressure (*p* = <0.001, rho = −0.256) and future prospects (*p* = 0.029, rho = −0.140). Lastly, general academic stress and each of its dimensions are positively and significantly related with each other (*p* = <0.01).

Table 4 presents the results of the Mann–Whitney U test for calculating the mean differences based on gender. Statistically significant differences were found in PA (*p* = 0.005, g = 0.71), with higher mean values in men. In turn, there are significant differences in general academic stress (*p* = 0.007, g = 0.79) and the dimension of academic overload (*p* = <0.001, g = 0.88), with higher mean scores for women. No significant statistical differences were present in the other variables; however, it is noteworthy that women present higher mean values in all dimensions of academic stress.

Lastly, the results of the Kruskal–Wallis H test in Table 5 show significant differences related to academic performance in general academic stress (H = 6.79, *p* = 0.034, η^2^ = 0.02) and in family pressure (H = 21.36, *p* = <0.001, η² = 0.08). After the post hoc analysis using the Games–Howell method, students with high academic performance present significantly lower mean scores than students with a medium (*p* = 0.002) and lower (*p* = <0.001) performance in the family pressure dimension. However, general academic stress did not exhibit significant differences according to low, medium, and high academic performance after the post hoc analysis.

## 4. Discussion

The main objective of this study was to analyze the relationships between academic performance, physical activity, and academic stress in secondary education students, while the secondary objective was to establish differences by gender of levels of physical activity and academic stress based on academic performance. This study shows an association between the variables analyzed, which provides key information for the acknowledgment of academic stress in the school context as a factor that affects the academic performance of students as multiple factors become stressors [25], for which effective coping strategies are required [26], such as the regular practice of PA [34,36], as students with higher levels of PA present less academic stress, and the lower the academic stress, the better the academic performance. In line with this, the findings of this study can lead to the design of effective coping strategies for school stress, with the goal of improving students’ academic performance through the practice of PA, which will contribute to the adherence of ESO students to a healthier lifestyle.

Initially, the descriptive data analysis reveals moderate PA levels in the population under study, a medium academic performance, and higher academic stress levels in the academic overload dimension, but lower ones in the interaction with classmate’s dimension. These results agree with those of previous studies showing that PA levels remain moderate–low, as students are a population that does not follow a great percentage of the established PA recommendations [10,12], due to the fact that they spend most of the day performing low-energy or sedentary activities, such as playing video games, watching television, or using the telephone, to which is added the little interest in encouraging young people to have a healthy and active lifestyle of the media that have a great influence on their behaviors [10,12].

Regarding academic performance, the relative mean of other studies often remains in the “medium” value scale, considering that there are multiple factors affecting this variable (economy, family, gender, sociodemographic characteristics) and that this, in turn, plays a mediating role in terms of healthy lifestyle habits [2,48]. In addition, in the works of Muñoz-Donoso et al. [6] and Borghi et al. [23], high academic stress levels from academic overload were also found, with physically less active students perceiving more stress from homework and assignments, and the exams period being frequently the biggest stressor in this category, mainly due to poor time management. Nevertheless, it is noteworthy that interaction with classmates motivates lower stress, as the pressure of the social environment has been demonstrated to also be a significant source of stress [26,49].

The results of the correlation analysis between variables indicate that both academic performance and PA are negatively associated with general academic stress, i.e., the higher the general academic stress, the lower the PA levels and academic performance. These findings are in line with studies such as that by Zhu et al. [15], which also demonstrates the need to reduce academic stress and increase PA practice, since the higher the academic stress levels, the lower the PA levels and the higher the vulnerability to mental health disorders. In this context, the study by Castro-Sánchez et al. [44] also shows that PA is associated with lower stress and higher academic performance, despite this last association not being significant in this study. This is because the regular practice of PA releases endorphins, which are neurotransmitters that generate feelings of happiness and reduce the perception of pain, which contributes to lower levels of anxiety and stress [44]. In addition, PA improves cognitive function, memory, and concentration, crucial factors for learning and academic performance [13,24].

In contrast, in the work by Castro-Sánchez et al. [44], academic performance also displays a positive relationship with stress; however, the authors warn that these data should be interpreted with caution, as is it understood that students with higher academic stress levels perform worse academically [50]. Regardless, students with medium levels of academic stress have been demonstrated to improve their learning achievements with adequate coping strategies, which can be, to a certain extent, an opportunity to improve performance [51,52]. Students can adapt to stressful situations thanks to a modulation in the production of cortisol, which provides the energy and substrates necessary to cope with the stimuli that give rise to stress [23], taking into account, in addition, its relationship with levels of motivation and resilience [23,44].

Regarding differences by gender, significant differences were found in PA levels, with higher mean values in men, which is in accordance with other studies where men presented the highest PA values [19,53]. In this line, Bobo-Arce et al. [54] and Burton et al. [55] believe that girls often have lower PA levels owing to different social stereotypes inside and outside the school environment that make them be more concerned with academic tasks. In addition, significant differences were observed in the general academic stress and the academic overload dimension, with higher mean values in women. This psychological malaise is attributed to the overload of academic tasks, assessments from teachers, classwork, and lack of time for academic-related activities [56]. In this sense, as indicated by Jones et al. [57], and Gasiūnienė and Miežienė [19], more physically active students—such as men in this case—present lower levels of academic stress. Therefore, the practice of regular PA is suggested to enrich the psychological health of students [58].

Lastly, with respect to academic performance, there are significant differences in general academic performance, and in the family pressure dimension, students who perform well academically present lower levels of academic stress derived from family pressure. In this context, the inverse relationship between family pressure and future prospects and academic performance found in the correlation analysis should be noted. Pressure from parents and the social environment has been demonstrated to increase the fear of failure, which may generate more anxiety and stress [26,49,59]. According to Naranjo [20], family pressure is classified as an external source of stress, while future prospects is an internal source; these sources increase in specific periods such as exam week, when the family’s perfectionist and rigid expectations exceed reality and generate frustration, making students feel incapable and unable to project their ideas for the future, and they arrives at a position of predominant anxiety. This scenario, according to Colunga-Rodríguez et al. [30], is associated with worse academic performance.

Thus, in line with the practice of PA as a potential coping strategy for academic stress, the study of its influence on academic performance should be furthered [60], underscoring the essential role of family in the promotion and adherence to PA, to which are attributed multiple benefits for mental health, such as the reduction of academic stress [61]. The social support provided by the family can significantly affect the motivation, participation, and continuity of young people in physical–sport activities, both positively and negatively, especially from an emotional perspective [20,59,62]. It has been shown that parents who adopt active behaviors and promote a healthy lifestyle tend to have children who also incorporate these habits into their daily lives [63]. Therefore, family support is essential to promote a physically active and healthy lifestyle during this stage, and it is even reflected in academic performance.

One of the main limitations of this study is its non-experimental and descriptive cross-sectional design, which does not allow for establishing causal relationships; therefore, the results should be interpreted with caution. In addition, regarding the measurement instruments, the use of accelerometers could provide more accurate information about the PA of students. Moreover, since all the participants attended public education centers, similar studies could be conducted in the future, but comparing public, state-subsidized, and private schools, expanding the sample size and including upper and lower grades to determine how the results change based on grade and age. Lastly, after analyzing the results of this study, interventions and longitudinal experimental studies should be conducted with a focus on the regular practice of PA, to measure its influence on the stress and academic performance of students.

## 5. Conclusions

With respect to the objectives proposed, and specifically to the main objective, it is concluded that academic performance, PA, and academic stress have different relationships with one another. In this context, academic performance is negatively associated with general academic stress, while general academic stress and each of its dimensions are positively related.

As for the secondary objective, ESO students see their PA levels and academic performance reduced as academic stress increases. In this scenario, women score higher in general academic stress and academic overload, and present lower levels of PA. Conversely, men present higher levels of PA and less academic stress in all dimensions. Therefore, it can be asserted that PA is an effective coping mechanism for academic stress disorder in secondary education. Lastly, based on academic performance, statistical differences are found in the family pressure dimension as a source of academic stress, with high-achieving students presenting less family pressure compared to students with a medium and low academic performance. Thus, the family plays a key role in the onset of stress. Therefore, encouraging the practice of PA among students should be a priority task for families and schools, proposing spaces and activities that are adapted to the needs and interests of students in order to improve their physical and mental health, which, in turn, can potentially contribute to better school performance.

## Figures and Tables

**Table 1:** Mean and standard deviation of the variables under study.

	M	SD
Physical activity	2.66	0.77
Academic performance	6.87	1.62
General academic stress	2.88	0.80
Academic overload	3.34	0.90
Interaction with classmates	2.38	0.89
Future prospects	2.87	1.16
Family pressure	2.93	1.04

Note: M = mean; SD = standard deviation.

**Table 2:** Descriptive analysis of academic performance.

	N	%
Low	93	30
Medium	144	46.4
High	73	23.6
Total	310	100

**Table 3:** Correlation coefficients between the study variables.

		2.	3.	4.	5.	6.	7.
1. Physical activity	Rho	0.087	−0.119	−0.150 *	−0.042	−0.025	−0.115
	Sig.	0.268	0.147	0.049	0.575	0.739	0.138
2. Academic performance	Rho		−0.169 *	−0.069	−0.046	−0.256 **	−0.140 *
	Sig.		0.016	0.293	0.467	<0.001	0.029
3. General academic stress	Rho			0.842 **	0.767 **	0.777 **	0.800 **
	Sig.			<0.001	<0.001	<0.001	<0.001
4. Academic overload	Rho				0.454 **	0.531 **	0.550 **
	Sig.				<0.001	<0.001	<0.001
5. Interaction with classmates	Rho					0.492 **	0.574 **
	Sig.					<0.001	<0.001
6. Family pressure	Rho						0.525 **
	Sig.						<0.001
7. Future prospects							

Note: * = *p* < 0.05; ** = *p* < 0.01; Rho = Spearman’s correlation coefficient.

**Table 4:** Statistical differences by gender.

Variables	Gender		*p*	G
	Man M ± SD	Women M ± SD		
Physical activity	2.77 ± 0.69	2.49 ± 0.74	0.005	0.71
Academic performance	6.86 ± 1.53	6.92 ± 1.73	0.478	1.63
General academic stress	2.75 ± 0.84	3.05 ± 0.71	0.007	0.79
Academic overload	3.15 ± 0.90	3.58 ± 0.84	<0.001	0.88
Interaction with classmates	2.28 ± 0.88	2.46 ± 0.83	0.051	0.86
Family pressure	2.86 ± 1.16	2.92 ± 1.16	0.728	1.16
Future prospects	2.88 ± 1.09	3.01 ± 0.98	0.252	1.05

Note: M = mean; SD = standard deviation; *p* = significance (<0.05); G = heel effect size.

**Table 5:** Statistical differences by academic performance.

Variables	Academic Performance			*p*	η²
	Low M ± SD	Medium M ± SD	High M ± SD		
Physical activity	2.54 ± 0.78	2.76 ± 0.73	2.55 ± 0.72	0.117	0.02
General academic stress	3.02 ± 0.80	2.85 ± 0.76	2.69 ± 0.80	0.034	0.02
Academic overload	3.45 ± 0.92	3.29 ± 0.89	3.28 ± 0.95	0.247	0.01
Interaction with classmates	2.30 ± 0.85	2.41 ± 0.86	2.14 ± 0.87	0.144	0.01
Family pressure	3.11 ± 1.07	2.86 ± 1.17	2.18 ± 1.10	<0.001	0.08
Future prospects	3.07 ± 1.10	2.79 ± 0.99	2.78 ± 1.06	0.123	0.02

Note: M = mean; SD = standard deviation; *p* = significance (<0.05); η² = effect size of square eta.

## Data Availability

Data are not available due to ethical requirements because they contain personal data.
